# Levels of NP and BPA in the Pearl River Estuary, China: Fluctuations with Country Policy Changes over the Past 40 Years

**DOI:** 10.3390/ijerph16214100

**Published:** 2019-10-24

**Authors:** Qi Chen, Yu Lan, Jian Shi, Weijie Liu, Bo Zhu, Dong Sun, Shunshan Duan

**Affiliations:** 1 Department of Ecology, Jinan University, Guangzhou 510632, China; cq92088@outlook.com (Q.C.); lanyu0706@163.com (Y.L.); sj150018@163.com (J.S.); jnu_sundong@163.com (D.S.); 2 South China Institute of Environmental Science, Ministry of Ecology and Environment of the People’s Republic of China, Guangzhou 510530, China; liuweijie_84@163.com; 3 School of Life Science and Engineering, State Defense Key Laboratory of the Nuclear Waste and Environmental Security, Southwest University of Science and Technology, Mianyang 621010, China; zzhubo@126.com

**Keywords:** nonylphenol, bisphenol A, dry mass sedimentation rates, historical trends, Pearl River Estuary

## Abstract

Sediment cores were collected from four outlets in the Pearl River Estuary (Guangdong Province, China) and dated using the ^210^Pb method to investigate the pollution history of the area due to its relatively stable sedimentation status and hydrographic conditions in recent decades. The ages of the sediment cores were dated over 40 years (1968–2015). The concentrations at the four outlets ranged from 2.21 to 48.52 ng g^−1^ dw for nonylphenol and were non-detectable for 23.64 ng g^−1^ dw for bisphenol A (BPA), which exhibited a decreasing trend from north to south as well as seaward. The fluxes (2.84 to 112.91 ng cm^−2^ yr^−1^ and non-detectable to 59.33 ng cm^−2^ yr^−1^ for nonylphenol and bisphenol A, respectively) stabilized in the 1980s to 1990s due to the construction of sewage treatment systems. The fluxes increased again in the 21st century, which reached a peak ca. 2010 but declined in recent years due to the establishment of regulations and the decreasing number of industrial enterprises. Fluctuations in the pollution composition coincided with industrial development and governmental policies.

## 1. Introduction

Endocrine-disrupting compounds (EDCs) have aroused significant concern in recent decades because of their potential adverse effects on the endocrine and reproductive systems of organisms [1]. Due to economic development and population growth in the 1970s, the increasing use of alkylphenol ethoxylates in industrial and household liquid detergents has been responsible for the anticipated steady growth of nonylphenol (NP) consumption [2]. 

Alkylphenol ethoxylates are widely used for cleaning formulations and as industrial process aids. Their wide applications include use as dispersing agents in paper and pulp production, emulsifying agents in paints and pesticide formulations, flotation agents, industrial cleaners (metal surfaces, textile processing, and the food industry), cold cleaners for cars, and household cleaners [3]. Approximately 80% of nonylphenol (NP) results from the degradation of alkylphenol ethoxylates, which are widely used in industrial, agricultural, and household applications, such as in liquid detergents. 

Bisphenol A (BPA) is used as an intermediate (binding, plasticizing, and hardening agent) in plastics, paints/lacquers, binding materials, and filling materials [4]. More than 90% of BPA is produced to make plastics, especially resins and polymers [5]. BPA production has grown over the past few years due to the demand for polycarbonates and resins [6]. NP and BPA are regarded as persistent toxic compounds by the United Nations Environment Program and are restricted by the European Chemicals Agency (ECHA) [7]. Even low concentrations of alkylphenols can lead to chronic toxicity in aquatic organisms, which threaten the ecological system balance due to the accumulation of EDCs in biota and the associated estrogenic-like effects. The widespread use and large-scale production of these compounds in many fields has also led to daily human exposure. Humans can take up these compounds through skin contact, respiration, and from food and water [8]. 

The global demand for BPA exceeded 6.5 million tons in 2012 and is predicted to increase at an annual rate of 4.6% from 2013 to 2019 [9]. For NP, the consumption in India showed an increasing trend, with the consumption of nonylphenol ethoxylates (NPEO) between 40 and 44 kilotons. By comparison, 25 to 50 kilotons of NP was consumed per year in the EU, but the usage of alkylphenol ethoxylates has declined as a result of self-regulation in European industries [10]. However, authorities in many countries lack monitoring and supervisory capabilities to restrict usage. NP and BPA are two typical EDCs with moderate estrogenic potency, but they have many more applications compared with other EDCs [11]. BPA is listed as an endocrine disruptor and has been proven to show estrogenic activity at low concentrations (<1 μg/m^3^). Moreover, it can exert toxic effects at low levels and can act as a neurodevelopmental toxicant [12,13]. NP was found to decrease the body weight and liver function of rats after exposure to 5 μg/day NP for 35 days [14].

NP and BPA have also been ubiquitously detected worldwide in water, food, soil, sediment, organisms, and even the atmosphere [13,15]. NP and BPA mainly drain into water through industrial wastewater discharge, and developed cities may exhibit high levels of contamination [16,17]. The high hydrophobicity of NP and BPA allows them to be easily deposited in sludge after draining into water, and their long half-lives under anaerobic sediment make them difficult to degrade (mean half-life of 301 days for NP and little or no degradation of BPA within 70 days) [18,19,20,21,22]. The observation of NP in aquatic microcosms demonstrates that the sediment is the key sink of NP deposition. Furthermore, the concentrations in the sediment have been found to increase with the associated aquatic concentration [4,23]. Wastewater treatment plants in cities can remove most alkylphenols, but, with the high consumption of alkylphenols and illegal discharge, large quantities of contaminates continue to drain into rivers and estuaries, especially in developing countries, and, thus, most alkylphenol accumulates in the sediment.

Many studies have focused on the distribution and occurrence of alkylphenols worldwide, such as in the air, water, sludge, and the organs of organisms [24,25,26,27]. Further studies have developed reconstructions of the pollution histories of various organic pollutants [28,29,30]. Although NP and BPA are widely used worldwide, their pollution histories and sources are still unclear in different regions and countries, including China, where environmental awareness began relatively late and historical records of contamination are lacking [31]. 

Most studies have examined surface sediment, which reflects only the current pollution status, and no information is available regarding the trends and sources in different areas. Table 1 shows partial studies regarding the pollution levels of NP and/or BPA in surface sediment worldwide. In this study, four sedimentary cores were collected from four different outlets of the Pearl River Estuary to investigate the pollution history at each outlet and to determine which outlet contributed the most to the pollution in past decades based on the sedimentation rates determined by using an appropriate model, concentrations’ measurement by liquid chromatograph-mass spectrometer (LC/MS), and fluxes of NP and BPA are combined with the developmental status and policies. Each outlet is associated with one or several cities that have different levels of industrial development but similar regulation policies in recent years. This work presents the current pollution trends, updates recent historical trends in the study areas, and attempts to determine the efficiency of regulation policies for NP and BPA.

## 2. Materials and Methods

### 2.1. Sediment Collection

**Figure 1:**
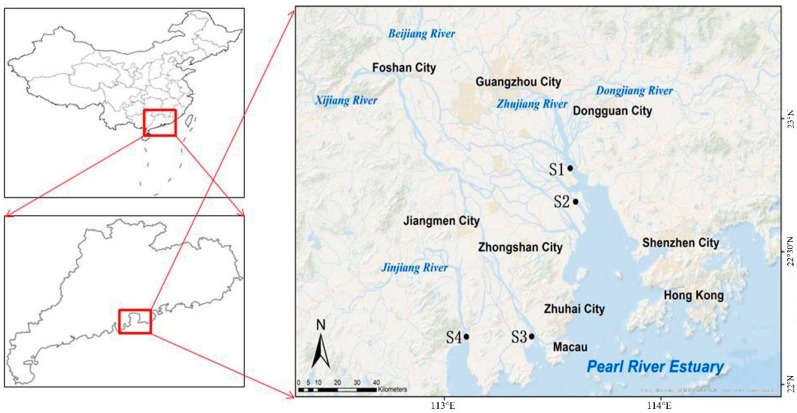
Map of the study areas and sampling sites. S1 was located in the Humen outlet (22°48.851′ N, 113°34.829′ E), S2 was located in the Jiaomen outlet (22°41.262′ N, 113°36.338′ E), S3 was located in the Modaomen outlet (22°10.927′ N, 113°24.231′ E), and S4 was located in the Yamen outlet (22°41.262′ N, 113°36.338′ E). The base map (sharing scope: all users, powered by Esri) was obtained in ArcGIS Online and edited by ArcGIS 10.2 (the URL for the base map: http://server.arcgisonline.com/arcgis/rest/services/Ocean/World_Ocean_Base/MapServer).

In the present study, four sediment cores, called S1, S2, S3, and S4, were taken from outlets in the Pearl River Estuary or the Pearl River Delta, the locations of which are shown in Figure 1. The samples were collected using a gravity core to minimize the sampling disturbance through careful experimental procedures or by assuming that the sediment had not been disturbed, as in similar studies [28,56,57,58]. The length of the samples was greater than 50 cm, and each column was sliced into 2-cm samples on-site using a stainless-steel cutting ring (diameter: 60 mm, height: 2 cm). Subsamples were packed in aluminium foil, placed in a refrigerator (−20 °C), and transported to the laboratory. All samples were stored at −20 °C until analysis.

### 2.2. Chemicals and Reagents

The 4-NP and BPA (99%) standards were purchased from Aladdin. HPLC-grade solvents were purchased from Anpel. The solid-phase extraction column (Sep-pak silica gel 500 mg/6 mL) was purchased from Waters. The filters were purchased from Whatman. All glassware was heated at 500 °C for 5 h. In addition, the use of plastic products was minimized as much as possible.

### 2.3. Extraction and LC/MS Analysis

The sediment samples were freeze-dried for at least 24 h. After drying, the samples were homogenized by sieving through a 100-mesh stainless-steel filter and stored in a dryer until analysis. To quantify the concentration, 1.0 g of the sample was dispersed in 10 mL of ethyl acetate using a vortex mixer for 30 s. Then, ultrasonic extraction was applied for 90 min to improve the compound extraction. The mixture was centrifuged for 15 min at 3500 rpm, and the supernatants were collected. This procedure was repeated twice. The final supernatants were filtered through a 0.45-μm filter and dried under a gentle stream of nitrogen. The mixture was dissolved in 2 mL of n-hexane before measuring the concentration. The SPE (solid-phase extraction) cartridges were successively pre-conditioned with 5 mL of methanol, 5 mL of ethyl acetate, and 5 mL of n-hexane. NP and BPA were eluted with 3 × 2 mL of ethyl acetate and 3 mL of dichloromethane. The eluents were blown dry by nitrogen, dissolved in 1 mL of methanol, passed through a 0.22-μm filter, and stored in an amber glass vial at −20 °C until analysis. Each sample was extracted three times.

The samples were analysed by liquid chromatography tandem mass spectrometry (4000 Q TRAP^®^ LC-MS/MS). More details are given in a previous publication [59].

### 2.4. Dry Density and Grain Size Measurements

The water content was measured using the method of Bian [47]. Clean and weighed stainless-steel specimen rings were used to collect the samples. Then, the wet and dry samples were weighed.

The dry density was measured using the following equations [30,60].

(1) $$wc=\frac{mw-md}{md}\times 100\%$$

where *wc* is the water content (%) and mw and md are the net masses of the wet and dry sediment, respectively.

(2) $$\rho_{sed}=\frac{\rho_{r}\times \rho_{w}}{wc\times \rho_{r}+\rho_{w}}$$

*ρ_sed_* is the density of the dry sediment (g cm^−3^), and *ρ_r_* and *ρ_w_* are the densities of rock (2.67 g cm^−3^) and water (1 g cm^−3^), respectively.

Grain size measurements were performed using a Malvern MS2000+2000MU (Malvern Panalytical, Malvern, UK). The samples were dried at 110 °C for 24 h. Then, metals were removed by 15 mL of 15% HCl, and dilute hydrogen peroxide was added to remove organic matter. Twenty milli-liters of NaOH (30%) was added to remove diatoms, and then 20 mL of sodium hexametaphosphate (0.5 mol/L) was added as a dispersant before measurement.

### 2.5. Chronological Method

Our dating method was based on those used in similar studies of adjacent areas and other estuaries with strong human activity, most of which investigated pollution histories in the environment [29,30,47,57,58,61,62,63,64]. Sedimentation rates were measured by the ^210^Pb method in the Department of Ecology, Jinan University. The activities of ^210^Pb and ^226^Ra were determined by a direct gamma assay using a high-purity germanium detector. Excess ^210^Pb was calculated by subtracting ^226^Ra from the total ^210^Pb. Due to the samples not reaching the ^226^Ra-^210^Pb radioactivity equilibrium depth, the constant initial concentration (CIC) model was used to derive the average dry mass sedimentation rate. To decrease vertical compaction, depth was replaced by mass depth, and the sedimentation rate was replaced by the mass accumulation rate or the dry mass sedimentation rate. The mass depth (*M*) was calculated by using the following equation.

(3) $$M=\overset{i}{\underset{0}{\int }}\left(1-\varphi \right)\rho_{sed}di$$

where *φ* is porosity in the sediment in layer *I* and *φ* is calculated by the water content.

(4) $$\varphi =\frac{wc}{wc+\frac{100-wc}{\rho_{sed}}}$$

General dry mass sedimentation rates were measured by ^210^Pb based on the CIC (constant initial concentration) model [29,65].

(5) $$t_{i}=1/\lambda \ln\left(A_{0}/A_{i}\right)$$

where *t_i_* is the time of year, *λ* is the decay constant of Pb, measured to be 0.03114 yr^−1^, *A*_0_ (Bq/kg) is the surface radioactivity, and *A_i_* (Bq/kg) is the radioactivity of layer *i*.

### 2.6. NP and BPA Fluxes

The NP and BPA concentrations in the sediments are reported in nanograms per gram of sediment in dry weight (ng g^−1^ dw). The flux was estimated using the following equation.

(6) $$Flux=c_{i}\times r_{i}$$

where *c_i_* (ng g^−1^ dw) and Cb are the measured and background concentrations of NP or BPA in the sediment layer *i*, respectively, and *r_i_* (g cm^−2^ yr^−1^) presents the dry mass sedimentation rate of sediment layer *i*, respectively.

### 2.7. Quality Assurance and Quality Control

Quality assurance and quality control were determined mainly as described in a previous study [59]. Extraction recovery was performed at three levels (10, 50, and 100 ng/L) using blank samples. Blank samples were prepared by heating the sediment samples at 500 °C for 5 h and then adding an injection standard of NP and BPA for the quality control. The average recoveries of NP and BPA in the sediment were 85.62% and 93.21%, respectively. The calibration curves exhibited good linear relationships (R^2^ > 0.99) between the limit of detection (LOD) and 200 ng/L for all samples. In this study, values below the limit of quantification (LOQ) are regarded as zero. The LOQs (limits of quantification) of NP and BPA were 0.27 and 0.28 ng g^−1^, respectively.

### 2.8. Data Analysis and Statistics

The values presented in the figures and tables are the means and standard deviations, which were determined in triplicate. Data visualization and correlation analysis were performed using OriginPro 2015. In addition, all industrial and anthropogenic data were collected from the Statistics Bureau of Guangdong Province (www.gdstats.gov.cn).

## 3. Results and Discussion

### 3.1. Chronology and Sediment Features

**Figure 2:**
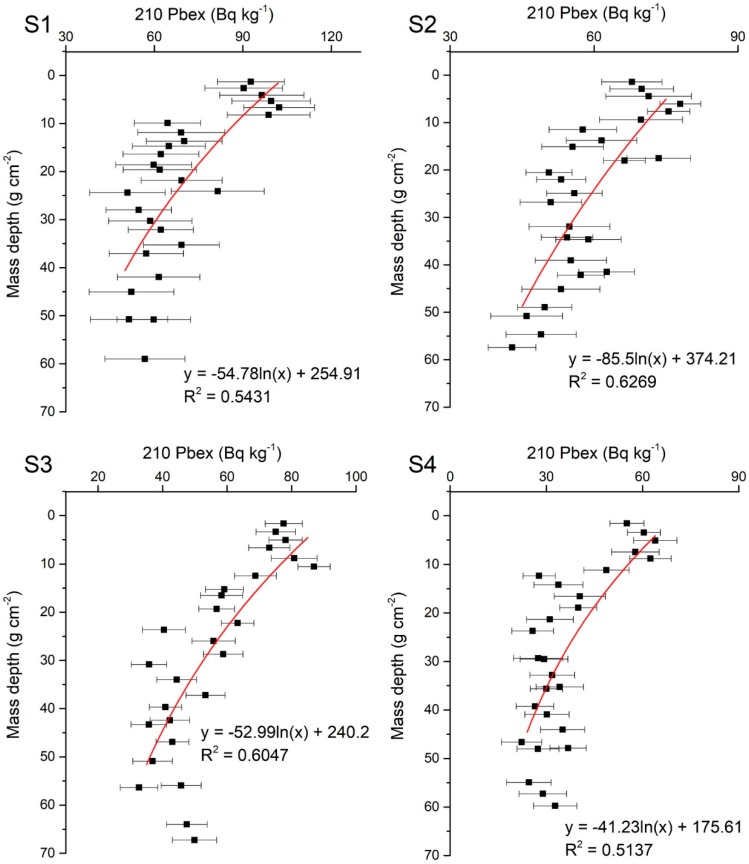
Radioactivity of excess ^210^Pb (^210^ Pb_ex_) in sampling sites. S1-4 present the sampling sites in Figure 1.

The dry mass sedimentation rates at the sample sites, which were calculated using the CIC model shown in Equations (4) and (5), are presented in Figure 2 and Table 2. A logarithmic plot of the ^210^Pb_ex_ activity displayed a general decrease in the sediment. The CIC model yielded mean dry mass sedimentation rates of 1.71, 2.66, 1.65, and 1.28 g cm^−2^ yr^−1^ for S1, S2, S3, and S4, respectively. The highest dry mass sedimentation rate was observed in S2, from the Jiaomen outlet, and the lowest rate was found in S4, from the Yamen outlet. The overall period ranged from 1968 to 2015: 1980 to 2015 in S1, 1993 to 2015 in S2, 1974 to 2015 in S3, and 1968 to 2015 in S4.

**Figure 3:**
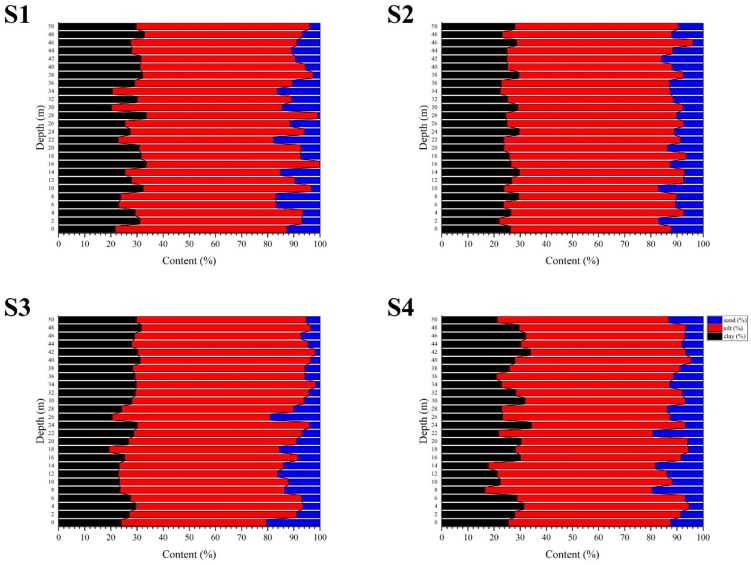
Profiles of the grain size in the sediment. S1-4 present the sampling sites in Figure 1.

The grain size compositions were relatively stable in the study areas (Figure 3). The main component of the sediments in these areas was silt, which accounted for 62.51%, 63.48%, 64.41%, and 63.33% of the compositions of S1, S2, S3, and S4, respectively.

Due to the effect of compaction, the water content and dry density of the sediment generally decreased from top to bottom (Figure S5). Previous research has reported similar decreasing trends in the water content and dry density [29,63,64]. The four cores consisted of relatively stable, fine-sized grains, and the ratios of clay to silt were constant, which were similar to those observed in a related study of the Pearl River Estuary [62,66,67]. Naturally, sediment composition is mainly determined by the water discharge and sediment load, which are influenced by rainfall. Since the 1950s, the water discharge in the Pearl River basin has remained constant except for a few droughts and floods [68]. In the present study, the stable ratio of clay and silt indicates a relatively stable dry mass sedimentation rate in each site. However, each outlet has a different contribution to the whole basin, which will be discussed below. Additionally, inordinate anthropogenic influences, such as desilting, dredging, and sand transport in the study areas, may have affected the original sediment [69].

The sediment cores in the study areas exhibited different dry mass sedimentation rates. S2 had the highest rate, and the dry mass sedimentation rate of S1 was lower than that of S2 but higher than those of S3 and S4. This result may be due to unbalanced regional development and different hydrological conditions. S1 was taken from the Humen outlet, which is composed of outflow from the Zhujiang River and Dongjiang River, and S2 was taken from the estuary by the Jiaomen outlet, which is comprised of the outflow from the Beijiang River and a minor part of the Xijiang River (Figure 1). The Humen and Jiaomen outlets comprised 9.3% and 18.2% of the sediment loads and 18.5% and 17.3% of the water discharge in the Pearl River Delta, respectively [68]. In addition, the sedimentation rate in the Pearl River Estuary ranges from 0.5 to 2.5 cm yr^−1^, as determined by a comparison with adjacent areas. The depositional environments among the four outlets are different but share some commonalities. The grain compositions in the outlets are similar (dominated by silt and clay) and have strong runoff from tributaries (runoff is stronger than tide). The water depth at the sampling sites is less than 5 m. Specifically, the sedimentation rates along the western and northern coasts were higher than those along the eastern coast because the western and northern coasts have more tributaries than the eastern coast [70]. In our study, S1 and S2 were collected from the western shore. The sedimentation rates are similar to those in adjacent areas in these cases, and the sedimentation rates in the Pearl River Estuary have been constant over 100 years, as determined by ^210^Pb dating of a few cores [62]. However, the sedimentation rates along the southern coast were determined based on limited data, which has prevented comparisons among studies. S3 and S4 were collected from the Modaomen outlet and the Yamen outlet, which are the main outflows of the Xijiang River and the Jinjiang River, respectively. S3 and S4 had lower sedimentation rates than S1 and S2, potentially because of the lowest water discharge and sediment load found at the Yamen outlet [68]. The highest water discharge and sediment load were found at the Modaomen outlet, but core S3 did not have the highest sedimentation rate, possibly due to reduced human activity in the Beijiang River and the Jinjiang River basins. The trend in the sedimentation rate exhibited a gradual decrease from north to south as well as seaward in the Pearl River Estuary [2,62]. Similar trends have also been found in the Yangtze River Estuary in China [29,63].

### 3.2. Concentrations and Distributions of NP and BPA

**Figure 4:**
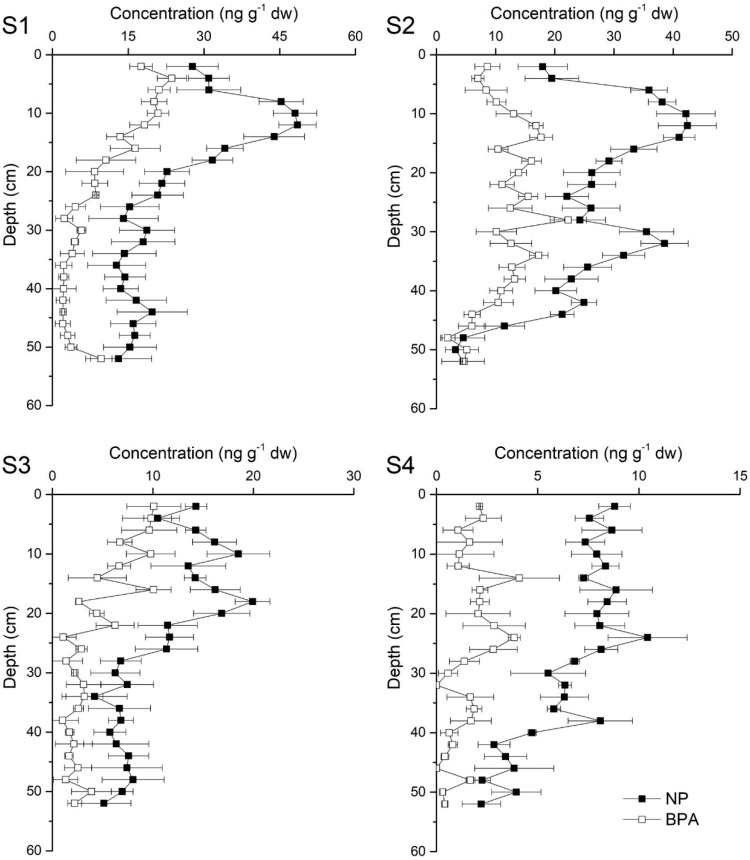
Vertical distributions of NP and BPA concentrations in the study areas. S1-4 present the sampling sites in Figure 1.

The concentrations of NP and BPA are shown in Table 2 and are illustrated in Figure 4. NP was detected in each layer. The NP concentrations ranged from 2.21 to 48.52 ng g^−1^ dw (mean value of 16.71 ng g^−1^ dw), and their fluxes ranged from 2.84 to 112.91 ng cm^−2^ yr^−1^. The BPA concentrations and fluxes ranged from non-detectable (LOQ) to 23.64 ng g^−1^ dw (mean value of 6.60 ng g^−1^ dw) and LOQ to 59.33 ng cm^−2^ yr^−1^, respectively. 

In the present study, the mean NP concentrations in the surface sediments (18.92 ng g^−1^ dw) were lower than those in the European countries in recent years (Table 1). In our investigation, they were also lower than those in most Asian countries but were comparable to those in some estuaries and lakes in Asia [42,47,48,49]. The NP concentrations in surface sediments were only higher than those in the river estuary around Dianchi Lake [50]. 

The BPA concentrations (10.12 ng g^−1^ dw) in the present study were lower than those in surface sediments in the Minho River, Iberian rivers (Spain), the Northern Aegean Sea (Greece), Mumbai (India), Anzali Wetland (Iran), the Klang River Estuary (Malaysia), the river estuary around Dianchi Lake (China), Daliao River Estuary (China), Halfmoon Bay Marina, Hobson Bay, and Milford Marina (New Zealand). The details are shown in Table 1. The BPA concentrations obtained in our study were higher than those found in the Romagna area (Italy), Sacca di Goro (Italy), Yeongil Bay (South Korea), and North America such as Lake Erie and the Mississippi Sound sediments and concentrations obtained by Chunyang et al. [45]. The concentrations were comparable to those of Japan, the Changjiang River Estuary, and the East China Sea [45,47].

Alkylphenols in aquatic environments are discharged from industrial wastewater treatment plants much more than from domestic sites [71], which means that industrial areas are a major source of pollution. Additionally, the concentrations of NP and BPA exhibited differences among the sampling sites but showed similar trends, including a decreasing trend seaward. Similar findings have been reported in other research, such as NP in the Daliao River Estuary [51] and BPA in the Yangtze River Estuary during the dry season [72]. These findings were likely caused by pollutants that were transported from narrow rivers or tributaries to large aquatic systems and, ultimately, diluted by being dispersed and deposited. However, the unbalanced development among the study areas should be a source of concern. The Pearl River Delta has been well established as an important economic and industrial center of China for hundreds of years, especially since the reform and opening-up policy. Moreover, cities in this area have their own specific features. Coincidentally, our results (Table 2) indicated that S1 and S2 suffered more severe pollution than S3 and S4. As shown in Figure 1, Guangzhou City (GZ) and Dongguan City (DG) discharge pollutants into the Pearl River Estuary through S1, Guangzhou City (GZ) and Foshan City (FS) through S2, Zhongshan City (ZS) and Zhuhai City (ZH) through S3, and Jiangmen City (JM) and the western rural area through S4. These cities contained almost half of the factories in the Guangdong Province in recent years (1999–2015) with GZ, FS, and DG contributing the largest numbers because of the reform and opening-up policy (Figure S1). Table 1 indicates that the concentrations of NP and BPA in surface sediment were lower than those found in many studies. Otherwise, they were relatively high in deeper sediments in our study, which could be attributed to policy regulations in recent years. This could reasonably explain why the sampling sites had different levels of pollutants.

### 3.3. Temporal Distributions and Fluxes of NP and BPA 

**Figure 5:**
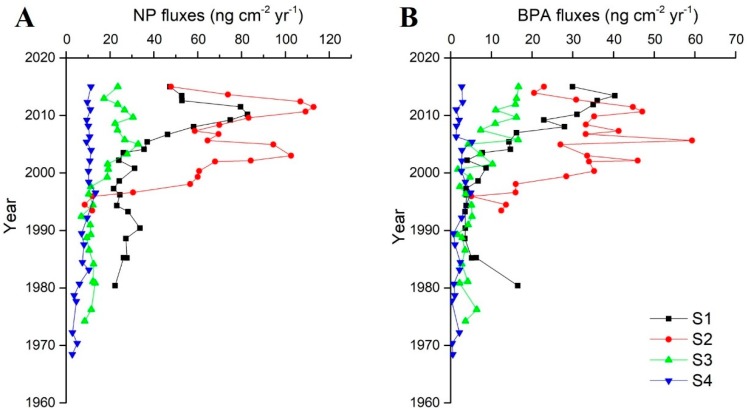
Fluxes of NP and BPA in the study areas. S1-4 present the sampling sites in Figure 1.

The fluxes of NP and BPA were calculated using Equation (6) and displayed the same trends as the concentrations (Figure 5). Alkylphenol fluxes could provide an index for the amounts of discharge and consumption during different periods. The flux of NP (2.84 to 112.91 ng cm^−2^ yr^−1^) was lower than those in Tokyo Bay, Japan (170 to 2770 ng cm^−2^ yr^−1^), Korea (18 to 159 ng cm^−2^ yr^−1^) [73,74], and the South China Sea (37–262 ng cm^−2^ yr^−1^) [2], but higher than that in the Yangtze River Estuary (0.68 to 17.9 ng cm^−2^ yr^−1^) [47]. The flux of BPA (LOQ to 59.33 ng cm^−2^ yr^−1^) was higher than those in the Yangtze River Estuary (0.62 to 3.13 ng cm^−2^ yr^−1^) and the South China Sea (<0.3 to 5.4 ng cm^−2^ yr^−1^) [2,47]. These higher fluxes indicate the cities near our study area that discharge more pollutants into the Pearl River Estuary.

The sediment cores in the sampling sites showed different trends but closely traced the historical economic development in the Pearl River Delta. Generally, the trends of alkylphenols in the Pearl River Estuary could be divided into several periods. (1) Before the 1980s and the initiation of the “reform and opening-up policy,” the fluxes of NP and BPA were lower than in the following period. (2) From the 1980s to the 1990s, the wastewater treatment plants’ (WWTP) construction project was established in China. This project might cause the discharge of alkylphenols to remain relatively stable. (3) The quantity of industry surged during the late 1990s to the late 2000s, which demanded more WWTP and new techniques when WWTP could not be satisfied. (4) The government paid considerable attention to environmental problems and enacted policies to restrict wastewater discharge standards and shut down a number of unqualified factories. Similar trends caused by anthropogenic activities (industrial and agricultural development, social policies such as a change in energy consumption and governmental policies) were found in NP and BPA as well as other organic pollutants, such as polycyclic aromatic hydrocarbons (PAHs), dichlorodiphenyl trichloroethanes (DDTs), and hexachlorocyclohexanes (HCHs) in China [28,29,30,47,63,75]. Some policies established in recent years should have effects, such as the “Strategy of Sustainable Development (SSD)” in 2003 and “The Construction of Ecological Civilization (CEC)” in 2012, where the Chinese government established restrictions on NP and NPEOs (Nonylphenol Ethoxylates) in 2011. These restrictions specified that NP and NPEOs can never be imported or exported, restricted BPA in baby bottles in 2006, charged for plastic bags in 2008, and limited BPA in food packages in 2011. In addition, the BPA in discarded plastic and resin material might leach into the environment [76]. The present results showed more complicated trends for BPA, which indicated that the unstable disposal of plastic and resin material may result in the fluctuation of BPA levels. More details of trends in S1, S2, and S3 are shown in the tables below (Table 3 and Table 4). The fluxes of the S4 core were not shown in the tables due to the inconspicuous variation in the fluxes, except that the fluxes after the mid-1980s were higher than before, which indicated that the reform and opening-up policy brought both economic development and a pollutant discharge.

Overall, the fluxes of NP and BPA were strongly impacted by the development of related industries and restrictive policies. However, the regional difference resulted in a different degree of policy implementation with regards to timeliness and effectiveness. Policies seemed more effective in major cities than in minor ones. The CEC policy impacted the pollutant trends where the strongest has an unprecedented requirement of water quality management and wastewater discharge through some indicators (total organic carbon, total nitrogen, and total phosphorus), which shut down many substandard factories to reduce pollutant emissions (Figures S2–S4). The environmental policies, development data, and trends shown in our study suggest that fewer pollutants are being discharged into the Pearl River Estuary.

## 4. Conclusions

Our study investigated historical pollution trends of NP and BPA in the Pearl River Estuary as well as the sedimentation rates of four outlets in this area. Our results indicate that the regulation policies have effectively reduced the discharge of contaminants. The vertical distributions of contaminants in the four outlets had similar features. The concentrations of NP were higher than those of BPA in all sampling sites, and the concentrations of NP and BPA generally decreased from the north to the south. According to the chronology study, the pollution status was the most serious from the beginning of the 21st century to approximately 2010, which coincided with economic development. Our data indicated that the Beijiang River contributed the majority of the pollution to the Pearl River Estuary, which proves that suggestions for regulating and monitoring based on anthropogenic data have had a significant impact on such pollutants.

## Figures and Tables

**Table 1:** Concentrations of NP and BPA in surface sediments (0–6 cm) from different sampling sites worldwide.

	Region	NP (ng g^−1^ dw)	BPA (ng g^−1^ dw)	References
Europe	Romagna area (North Italy)	25	3.9	[32]
	Atlantic coast (Andalusia, SW Spain)	127.6	NA	[33]
	Minho River	9.3–74.5	4.3–130	[34]
	Sacca di Goro (Italy)	326–424	<2.5–4.2	[35]
	Iberian rivers (Spain)	LOQ–1693	LOQ–117	[36]
	Northern Aegean Sea (Greece)	223–2695 (956)	7.2–39 (16.1)	[37]
	Southern Baltic Sea	2.7–1001	<0.5–<5.2	[38]
Asia	Outlets of Pearl River Estuary (China)	18.92	10.12	Present study
	Mumbai (India)	356.5	25.15	[39]
	Kaohsiung Harbour (Taiwan)	18–27882 (1101 ± 3580)	NA	[40]
	Anzali Wetland (Iran)	29000	7000	[41]
	Lake Shihwa (Korea)	16.1–21.5	NA	[42]
	Klang River Estuary (Malaysia)	NA	23.64	[43]
	Mangrove ecosystems (Singapore)	<0.4–81	NA	[44]
	Japan	NA	1.88–23.0 (8.17)	[45]
	Korea	NA	LOQ–13370	[45]
	East China Sea inner shelf (China)	31.3–1423.7 (750.1)	NA	[20]
	Yellow Sea (China)	349.5–1642.8 (890.1)	NA	[20]
	Lanzhou Reach of the Yellow River (China)	38.4–863.0	NA	[46]
	Changjiang River Estuary and East China Sea (China)	1.56–35.8	0.72–13.2	[47]
	Yeongil Bay (South Korea)	12.3–38.6	<1	[48]
	Taihu Lake (China)	20.66	NA	[49]
	River estuary around Dianchi Lake (China)	2–18 (7)	16–849 (165)	[50]
	Daliao River Estuary (China)	1.5–456	3.4–25.3	[51]
South America	Buenos Aires (Argentina)	7–3357	NA	[52]
North America	Lake Erie (the USA)	NA	6.1	[53]
	The USA	NA	LOQ–106 (5.14)	[45]
	Mississippi Sound sediments (the USA)	NA	LOQ–2.99	[54]
Oceania	Halfmoon Bay Marina, Hobson Bay, and Milford Marina (New Zealand)	NA	50,52,145	[55]

The values in brackets (xx.xx) stand for the mean value. LOQ stands for non-detectable. NA stands for not analyzed.

**Table 2:** Concentrations and fluxes of NP and BPA and time periods at sampling sites.

Core	Length (cm)	Time Period	Concentration (ng g^−1^ dw) NP BPA		Fluxes (ng cm^−2^ yr^−1^) NP BPA	
S1	52	1980–2015	12.71–48.52 (24.02)	2.05–23.64 (9.15)	21.68–82.77	3.49–40.33
S2	52	1993–2015	3.21–42.41 (25.72)	1.91–22.28 (11.32)	8.54–112.91	5.09–59.33
S3	52	1974–2015	5.13–19.93 (10.55)	1.01–10.10 (4.36)	8.47–32.88	1.67–16.66
S4	52	1968–2015	2.21–10.43 (6.53)	LOQ–4.09 (1.57)	2.84–13.39	LOQ–5.25
Summary		1968–2015	2.21–48.52 (16.71)	LOQ–23.64 (6.60)	2.84–112.91	LOQ–59.33

The values in brackets (xx.xx) stand for the mean value. LOQ stands for non-detectable.

**Table 3:** The main features of the sedimentary records of the fluxes of NP.

NP Flux			
Core	Peak time	Subsequent trend	Possible explanation
S1	1991	Decreased and then remained stable	Reform and opening-up policy ^a^. WWTP construction project completed ^b^.
	2000	Decreased and quickly bounced	Insufficient WWTP and techniques ^a^. SSD established in 2003 but QF (GZ, DG) still increased and QF (DG) jumped in 2005 ^b^.
	2010	Decreased	QF (GZ, DG) increased to a high level ^a^. Import and export restriction of NP, CEC established ^b^.
S2	2002	Decreased	QF (GZ, FS) increased, while WWTP were still insufficient ^a^. SDD established in 2003 ^b^.
	2011	Decreased	QF (GZ, DG) reached a high level ^a^. QF (GZ, FS) decreased from 2010 to 2011. CEC established ^b^.
S3	2005	Decreased	QF (ZS) increased quickly ^a^. SSD established ^b^.
	2009	Decreased	QF (ZH, ZS) reached the highest level ^a^. QF (ZH, ZS) decreased from 2010. CEC established ^b^.

QF presents the quantities of the industry, and details are shown in Figures S2 and S3. ^a^ A possible explanation of the peak time. ^b^ A possible explanation of the subsequent trend. WWTP presents wastewater treatment plants. SSD presents the “Strategy of Sustainable Development” policy. Abbreviations in bracket stands for cities which were defined above.

**Table 4:** Main features of the sedimentary records of the fluxes of BPA.

BPA Flux			
Core	Peak time	Subsequent trend	Possible explanation
S1	2000	Decreased and then bounced	Insufficient WWTP and techniques ^a^. SSD established in 2003 ^b^.
	2007	Decreased and then bounced	PF (DG) increased quickly until 2008 ^a^. Plastic bags require payments. BPA consumption decreased. PF (GZ, DG) jumped to the highest level in 2009 ^b^.
	2013	Decreased	PF (GZ, DG) increased again from 2011 ^a^. CEC established in 2012 ^b^.
S2	2000	Decreased and then bounced	PF (GZ) was stable, while PF (FS) started growing ^a^.
	2002	Decreased	PF (GZ, FS) kept increasing ^a^. SDD established ^b^.
	2005	Decreased to fluctuation	Fastest increasing rate of PF (FS) and exceeding level of PF (GZ) ^a^.
	2010	Decreased	PF (GZ, FS) reached a high level from 2008 to 2010 ^a^. PF (GZ, FS) decreased, BPA was banned in food packages, and CEC was established ^b^.
S3	2001	Decreased	PF (ZH) kept increasing ^a^. SDD was established in 2003 ^b^.
	2005	Decreased and then bounced	PF (ZS) increased quickly ^a^. Unknown reason for the decrease, while PF (ZH, ZS) kept increasing ^b^.
	2009	Decreased and then kept stable	PF (ZH, ZS) reached a high level ^a^. PF (ZH, ZS) decreased from 2010 and remained stable. CEC was established in 2012 ^b^.

PF presents the quantities of the plastic and rubber industries, and details are shown in Figures S2 and S3. ^a^ possible explanation of the peak time. ^b^ A possible explanation of the subsequent trend. CEC presents the “Construction of Ecological Civilization” policy. Abbreviations in bracket stands for cities which were defined above.

## Supplementary Materials

The following are available online at https://www.mdpi.com/1660-4601/16/21/4100/s1, Figure S1: Proportions of light industry (a), heavy industry (b) and plastic and rubber industry (c) among six cities, Figure S2. Trends of light industry enterprise quantity among six cities, Figure S3. Trends of heavy industry enterprise quantity among six cities, Figure S4. Trends of plastic and rubber industry enterprise quantity among six cities, Figure S5. Profiles of water content in sediment.

## References

[1] Diamanti-Kandarakis E., Bourguignon J.P., Giudice L.C., Hauser R., Prins G.S., Soto A.M., Zoeller R.T., Gore A.C. (2009). Endocrine-disrupting chemicals: An Endocrine Society scientific statement. Endocr. Rev..

[2] Peng X., Wang Z., Mai B., Chen F., Chen S., Tan J., Yu Y., Tang C., Li K., Zhang G. (2007). Temporal trends of nonylphenol and bisphenol A contamination in the Pearl River Estuary and the adjacent South China Sea recorded by dated sedimentary cores. Sci. Total Environ..

[3] Thiele B., Günther K., Schwuger M.J. (1997). Alkylphenol Ethoxylates: Trace Analysis and Environmental Behavior. Chem. Rev..

[4] Careghini A., Mastorgio A.F., Saponaro S., Sezenna E. (2015). Bisphenol A, nonylphenols, benzophenones, and benzotriazoles in soils, groundwater, surface water, sediments, and food: A review. Environ. Sci. Pollut. Res..

[5] Huang Y.Q., Wong C.K., Zheng J.S., Bouwman H., Barra R., Wahlstrom B., Neretin L., Wong M.H. (2012). Bisphenol A (BPA) in China: A review of sources, environmental levels, and potential human health impacts. Environ. Int..

[6] Flint S., Markle T., Thompson S., Wallace E. (2012). Bisphenol A exposure, effects, and policy: A wildlife perspective. J. Environ. Manag..

[7] UNEP (2002). Regionally Based Assessment of Persistent Toxic Substances—South East Asia and South Pacific.

[8] Kabir E.R., Rahman M.S., Rahman I. (2015). A review on endocrine disruptors and their possible impacts on human health. Environ. Toxicol. Pharmacol..

[9] Research T.M. (2013). Bisphenol A Market for Polycarbonates, Epoxy Resins and Other Applications—Global Industry Analysis, Aize, Share, Growth and Forecast, 2013–2019.

[10] Acir I.H., Guenther K. (2018). Endocrine-disrupting metabolites of alkylphenol ethoxylates—A critical review of analytical methods, environmental occurrences, toxicity, and regulation. Sci. Total Environ..

[11] Li X.L., Luan T.G., Liang Y., Wong M.H., Lan C.Y. (2007). Distribution patterns of octylphenol and nonylphenol in the aquatic system at Mai Po Marshes Nature Reserve, a subtropical estuarine wetland in Hong Kong. J. Environ. Sci. China.

[12] Rykowska I., Wasiak W. (2006). Properties, threats, and methods of analysis of bisphenol A and its derivatives. Acta Chromatogr..

[13] Michalowicz J. (2014). Bisphenol A—Sources, toxicity and biotransformation. Environ. Toxicol. Pharmacol..

[14] Kazemi S., Kani S.N.M., Ghasemi-Kasman M., Aghapour F., Khorasani H., Moghadamnia A.A. (2016). Nonylphenol induces liver toxicity and oxidative stress in rat. Biochem. Biophis. Res. Commun..

[15] Omar T.F.T., Ahmad A., Aris A.Z., Yusoff F.M. (2016). Endocrine disrupting compounds (EDCs) in environmental matrices: Review of analytical strategies for pharmaceuticals, estrogenic hormones, and alkylphenol compounds. TrAC Trends Anal. Chem..

[16] Cladiere M., Gasperi J., Lorgeoux C., Bonhomme C., Rocher V., Tassin B. (2013). Alkylphenolic compounds and bisphenol A contamination within a heavily urbanized area: Case study of Paris. Environ. Sci. Pollut. Res..

[17] Suzuki T., Nakagawa Y., Takano I., Yaguchi K., Yasuda K. (2004). Environmental fate of bisphenol A and its biological metabolites in river water and their xeno-estrogenic activity. Environ. Sci. Technol..

[18] Ying G.G., Williams B., Kookana R. (2002). Environmental fate of alkylphenols and alkylphenol ethoxylates—A review. Environ. Int..

[19] Isobe T., Nishiyama H., Nakashima A., Takada H. (2001). Distribution and behavior of nonylphenol, octylphenol and nonylphenol monoethoxylate in Okyo metropolitan area: Their association with aquatic particles and sedimentary distributions. Environ. Sci. Technol..

[20] Duan X.Y., Li Y.X., Li X.G., Zhang D.H., Gao Y. (2014). Alkylphenols in surface sediments of the Yellow Sea and East China Sea inner shelf: Occurrence, distribution and fate. Chemosphere.

[21] Venkatesan A.K., Halden R.U. (2013). National inventory of alkylphenol ethoxylate compounds in U.S. sewage sludges and chemical fate in outdoor soil mesocosms. Environ. Pollut..

[22] Ying G.G., Kookana R.S. (2003). Degradation of five selected endocrine-disrupting chemicals in seawater and marine sediment. Environ. Sci. Technol..

[23] Jin S.W., Yang F.X., Xu Y., Dai H.P., Liu W.P. (2013). Risk assessment of xenoestrogens in a typical domestic sewage-holding lake in China. Chemosphere.

[24] Meesters R.J.W., Schroder H.F. (2002). Simultaneous determination of 4-nonylphenol and bisphenol a in sewage sludge. Anal. Chem..

[25] Cai Y., Jiang G., Liu J., Liang X., Yao Z., Liu J., Liu J., Zhou Q. (2007). Solid-Phase Microextraction Coupled with High Performance Liquid Chromatography-Fluorimetric Detection for the Determination of Bisphenol A, 4-n-Nonylphenol, and 4-tert-Octylphenol in Environmental Water Samples. Anal. Lett..

[26] Inoue K., Yoshida S., Nakayama S., Ito R., Okanouchi N., Nakazawa H. (2006). Development of stable isotope dilution quantification liquid chromatography-mass spectrometry method for estimation of exposure levels of bisphenol A, 4-tert-octylphenol, 4-nonylphenol, tetrabromobisphenol A, and pentachlorophenol in indoor air. Arch. Environ. Contam. Toxicol..

[27] Geens T., Neels H., Covaci A. (2012). Distribution of bisphenol-A, triclosan and n-nonylphenol in human adipose tissue, liver and brain. Chemosphere.

[28] Yang Z.F., Tang Z.W., Shen Z.Y., Niu J.F., Wang H.Y. (2011). One-Hundred-Year Sedimentary Record of Polycyclic Aromatic Hydrocarbons in Urban Lake Sediments from Wuhan, Central China. Water Air Soil Pollut..

[29] Cai Y.Z., Wang X.H., Wu Y.L., Li Y.Y., Ya M.L. (2016). Over 100-year sedimentary record of polycyclic aromatic hydrocarbons (PAHs) and organochlorine compounds (OCs) in the continental shelf of the East China Sea. Environ. Pollut..

[30] Zhang R., Zhang F., Zhang T.C. (2013). Sedimentary records of PAHs in a sediment core from tidal flat of Haizhou Bay, China. Sci. Total Environ..

[31] Hu J., Wan Y., Shao B., Jin X., An W., Jin F., Yang M., Wang X., Sugisaki M. (2005). Occurrence of trace organic contaminants in Bohai Bay and its adjacent Nanpaiwu River, North China. Mar. Chem..

[32] Emanuela P., Enrico D. (2018). Distribution and partition of endocrine disrupting compounds in water and sediment: Case study of the Romagna area (North Italy). J. Geochem. Explor..

[33] Pintado-Herrera M.G., Combi T., Corada-Fernández C., González-Mazo E., Lara-Martín P.A. (2017). Occurrence and spatial distribution of legacy and emerging organic pollutants in marine sediments from the Atlantic coast (Andalusia, SW Spain). Sci. Total Environ..

[34] Salgueiro-González N., Turnes-Carou I., Besada V., Muniategui-Lorenzo S., López-Mahía P., Prada-Rodríguez D. (2015). Occurrence, distribution and bioaccumulation of endocrine disrupting compounds in water, sediment and biota samples from a European river basin. Sci. Total. Environ..

[35] Casatta N., Mascolo G., Roscioli C., Viganò L. (2015). Tracing endocrine disrupting chemicals in a coastal lagoon (Sacca di Goro, Italy): Sediment contamination and bioaccumulation in Manila clams. Sci. Total. Environ..

[36] Gorga M., Insa S., Petrovic M., Barceló D. (2015). Occurrence and spatial distribution of EDCs and related compounds in waters and sediments of Iberian rivers. Sci. Total Environ..

[37] Anastasia A., Dimitra V. (2012). Occurrence and partitioning of endocrine-disrupting compounds in the marine environment of Thermaikos Gulf, Northern Aegean Sea, Greece. Mar. Pollut. Bull..

[38] Ruczyå Ska W., Szlinder-Richert J., Drgas A. (2016). The occurrence of endocrine disrupting compounds in off-shore sediments from the southern Baltic Sea. Environ. Sci. Process Impacts.

[39] Tiwari M., Sahu S.K., Pandit G.G. (2016). Distribution and estrogenic potential of endocrine disrupting chemicals (EDCs) in estuarine sediments from Mumbai, India. Environ. Sci. Pollut. Res..

[40] Dong C.D., Chen C.W., Chen C.F. (2015). Seasonal and spatial distribution of 4-nonylphenol and 4-tert-octylphenol in the sediment of Kaohsiung Harbor, Taiwan. Chemosphere.

[41] Mortazavi S., Bakhtiari A.R., Sari A.E., Bahramifar N., Rahbarizade F. (2012). Phenolic endocrine disrupting chemicals (EDCs) in Anzali Wetland, Iran: Elevated concentrations of 4-nonylphenol, octhylphenol and bisphenol A. Mar. Pollut. Bull..

[42] Hong S., Won E.J., Ju H.J., Kim M.S., Shin K.H. (2010). Current nonylphenol pollution and the past 30 years record in an artificial Lake Shihwa, Korea. Mar. Pollut. Bull..

[43] Omar T.F.T., Aris A.Z., Yusoff F.M., Mustafa S. (2018). Occurrence, distribution, and sources of emerging organic contaminants in tropical coastal sediments of anthropogenically impacted Klang River estuary, Malaysia. Mar. Pollut. Bull..

[44] Bayen S., Estrada E.S., Juhel G., Kit L.W., Kelly B.C. (2016). Pharmaceutically active compounds and endocrine disrupting chemicals in water, sediments and mollusks in mangrove ecosystems from Singapore. Mar. Pollut. Bull..

[45] Chunyang L., Fang L., Hyo-Bang M., Nobuyoshi Y., Sehun Y., Kurunthachalam K. (2012). Bisphenol analogues in sediments from industrialized areas in the United States, Japan, and Korea: Spatial and temporal distributions. Environ. Sci. Technol..

[46] Xu J., Wang P., Guo W.F., Dong J.X., Wang L., Dai S.G. (2006). Seasonal and spatial distribution of nonylphenol in Lanzhou Reach of Yellow River in China. Chemosphere.

[47] Bian H.Y., Li Z.Y., Liu P., Pan J.F. (2010). Spatial distribution and deposition history of nonylphenol and bisphenol A in sediments from the Changjiang River (Yangtze River) Estuary and its adjacent East China Sea. Acta Oceanol. Sin..

[48] Koh C.H., Khim J.S., Villeneuve D.L., Kannan K., Giesy J.P. (2006). Characterization of trace organic contaminants in marine sediment from Yeongil Bay, Korea: 2. Dioxin-like and estrogenic activities. Environ. Pollut..

[49] Liu D., Liu J., Guo M., Xu H., Zhang S., Shi L., Yao C. (2016). Occurrence, distribution, and risk assessment of alkylphenols, bisphenol A, and tetrabromobisphenol A in surface water, suspended particulate matter, and sediment in Taihu Lake and its tributaries. Mar. Pollut. Bull..

[50] Wang B., Huang B., Jin W., Zhao S.M., Li F.R., Hu P., Pan X.J. (2013). Occurrence, distribution, and sources of six phenolic endocrine disrupting chemicals in the 22 river estuaries around Dianchi Lake in China. Environ. Sci. Pollut. Res..

[51] Li Z.Y., Gibson M., Liu C., Hu H. (2013). Seasonal variation of nonylphenol concentrations and fluxes with influence of flooding in the Daliao River Estuary, China. Environ. Monit. Assess..

[52] Babay P.A., Itria R.F., Ale E.E.R., Becquart E.T., Gautier E.A. (2014). Ubiquity of Endocrine Disruptors Nonylphenol and Its Mono- and Di-Ethoxylates in Freshwater, Sediments, and Biosolids Associated with High and Low Density Populations of Buenos Aires, Argentina. Clean Soil Air Water.

[53] Lu Z., Letcher R.J., Chu S., Ciborowski J.J.H., Haffner G.D., Drouillard K.G., Macleod S.L., Marvin C.H. (2015). Spatial distributions of polychlorinated biphenyls, polybrominated diphenyl ethers, tetrabromobisphenol A and bisphenol A in Lake Erie sediment. J. Great Lakes Res..

[54] Guangdi W., Peng M., Qiang Z., John L., Michelle L., Yoko F., O’Reilly S.E., Shelley M., John M.L., Shaoyuan Z. (2012). Endocrine disrupting chemicals in New Orleans surface waters and Mississippi Sound sediments. J. Environ. Monit. JEM.

[55] Stewart M., Olsen G., Hickey C.W., Ferreira B., Jelic A., Petrovic M., Barcelo D. (2014). A survey of emerging contaminants in the estuarine receiving environment around Auckland, New Zealand. Sci. Total Environ..

[56] Covelli S., Fontolan G., Faganeli J., Ogrinc N. (2006). Anthropogenic markers in the Holocene stratigraphic sequence of the Gulf of Trieste (northern Adriatic Sea). Mar. Geol..

[57] Bixian M., Zeng E.Y., Xiaojun L., Qingshu Y., Gan Z., Xiangdong L., Guoying S., Jiamo F. (2005). Abundances, depositional fluxes, and homologue patterns of polychlorinated biphenyls in dated sediment cores from the Pearl River Delta, China. Environ. Sci. Technol..

[58] Gan Z., Andrew P., Alan H., Bixian M., Xiangdong L., Yuehui K., Zhishi W. (2002). Sedimentary records of DDT and HCH in the Pearl River Delta, South China. Environ. Sci. Technol..

[59] Diao P., Chen Q., Wang R., Sun D., Cai Z., Wu H., Duan S. (2017). Phenolic endocrine-disrupting compounds in the Pearl River Estuary: Occurrence, bioaccumulation and risk assessment. Sci. Total Environ..

[60] Gouleau D., Jouanneau J.M., Weber O., Sauriau P.G. (2000). Short- and long-term sedimentation on Montportail-Brouage intertidal mudflat, Marennes-Oleron Bay (France). Cont. Shelf Res..

[61] Zhang Y., Lu X., Shao X., Liu H., Xing M., Zhao F., Li X., Yuan M. (2015). Influence of Sedimentation Rate on the Metal Contamination in Sediments of Bohai Bay, China. Bull. Environ. Contam. Toxicol..

[62] Qi S., Leipe T., Rueckert P., Di Z., Harff J. (2010). Geochemical sources, deposition and enrichment of heavy metals in short sediment cores from the Pearl River Estuary, Southern China. J. Mar. Syst..

[63] Lin T., Nizzetto L., Guo Z.G., Li Y.Y., Li J., Zhang G. (2016). DDTs and HCHs in sediment cores from the coastal East China Sea. Sci. Total Environ..

[64] Graca B., Staniszewska M., Zakrzewska D., Zalewska T. (2016). Reconstruction of the pollution history of alkylphenols (4-tert-octylphenol, 4-nonylphenol) in the Baltic Sea. Environ. Sci. Pollut. Res..

[65] Andersen T.J., Mikkelsen O.A., Moller A.L., Pejrup M. (2000). Deposition and mixing depths on some European intertidal mudflats based on Pb-210 and Cs-137 activities. Cont. Shelf Res..

[66] Zhong Y., Chen Z., Li L., Liu J., Li G., Zheng X., Wang S., Mo A. (2017). Bottom water hydrodynamic provinces and transport patterns of the northern South China Sea: Evidence from grain size of the terrigenous sediments. Cont. Shelf Res..

[67] Li G., Yan W., Zhong L.F. (2016). Element geochemistry of offshore sediments in the northwestern South China Sea and the dispersal of Pearl River sediments. Prog. Oceanogr..

[68] Wu C.S., Yang S.L., Huang S.C., Mu J.B. (2016). Delta changes in the Pearl River estuary and its response to human activities (1954–2008). Quat. Int..

[69] Zhao G., Ye S., Yuan H., Ding X., Wang J. (2017). Surface sediment properties and heavy metal pollution assessment in the Pearl River Estuary, China. Environ. Sci. Pollut. Res. Int..

[70] Ip C.C.M., Li X.D., Zhang G., Farmer J.G., Wai O.W.H., Li Y.S. (2004). Over one hundred years of trace metal fluxes in the sediments of the Pearl River Estuary, South China. Environ. Pollut..

[71] Lee S., Liao C., Song G.J., Ra K., Kannan K., Moon H.B. (2015). Emission of bisphenol analogues including bisphenol A and bisphenol F from wastewater treatment plants in Korea. Chemosphere.

[72] Shi J.H., Liu X.W., Chen Q.C., Zhang H. (2014). Spatial and seasonal distributions of estrogens and bisphenol A in the Yangtze River Estuary and the adjacent East China Sea. Chemosphere.

[73] Yamashita N., Kannan K., Imagawa T., Villeneuve D.L., Hashimoto S., Miyazaki A., Giesy J.P. (2000). Vertical profile of polychlorinated dibenzo-p-dioxins, dibenzofurans, naphthalenes, biphenyls, polycyclic aromatic hydrocarbons, and alkylphenols in a sediment core from Tokyo Bay, Japan. Environ. Sci. Technol..

[74] Moon H.-B., Choi M., Choi H.-G., Ok G., Kannan K. (2009). Historical trends of PCDDs, PCDFs, dioxin-like PCBs and nonylphenols in dated sediment cores from a semi-enclosed bay in Korea: Tracking the sources. Chemosphere.

[75] Peng X., Xiong S., Ou W., Wang Z., Tan J., Jin J., Tang C., Liu J., Fan Y. (2017). Persistence, temporal and spatial profiles of ultraviolet absorbents and phenolic personal care products in riverine and estuarine sediment of the Pearl River catchment, China. J. Hazard. Mater..

[76] Petrie B., Lopardo L., Proctor K., Youdan J., Barden R., Kasprzyk-Hordern B. (2019). Assessment of bisphenol-A in the urban water cycle. Sci. Total Environ..

