# Supplementary material for: Levels of NP and BPA in the Pearl River Estuary, China: Fluctuations with Country Policy Changes over the Past 40 Years
Source: Int J Environ Res Public Health. 2019 Oct 24;16(21):4100. doi: 10.3390/ijerph16214100 (PMC6862134; doi:10.3390/ijerph16214100)
Supplement: Supplementary file 1 [file ijerph-16-04100-s001.pdf]

Supplementary: Levels of NP and BPA in the Pearl River Estuary, China: Fluctuations with country policy changes over the past 40 years

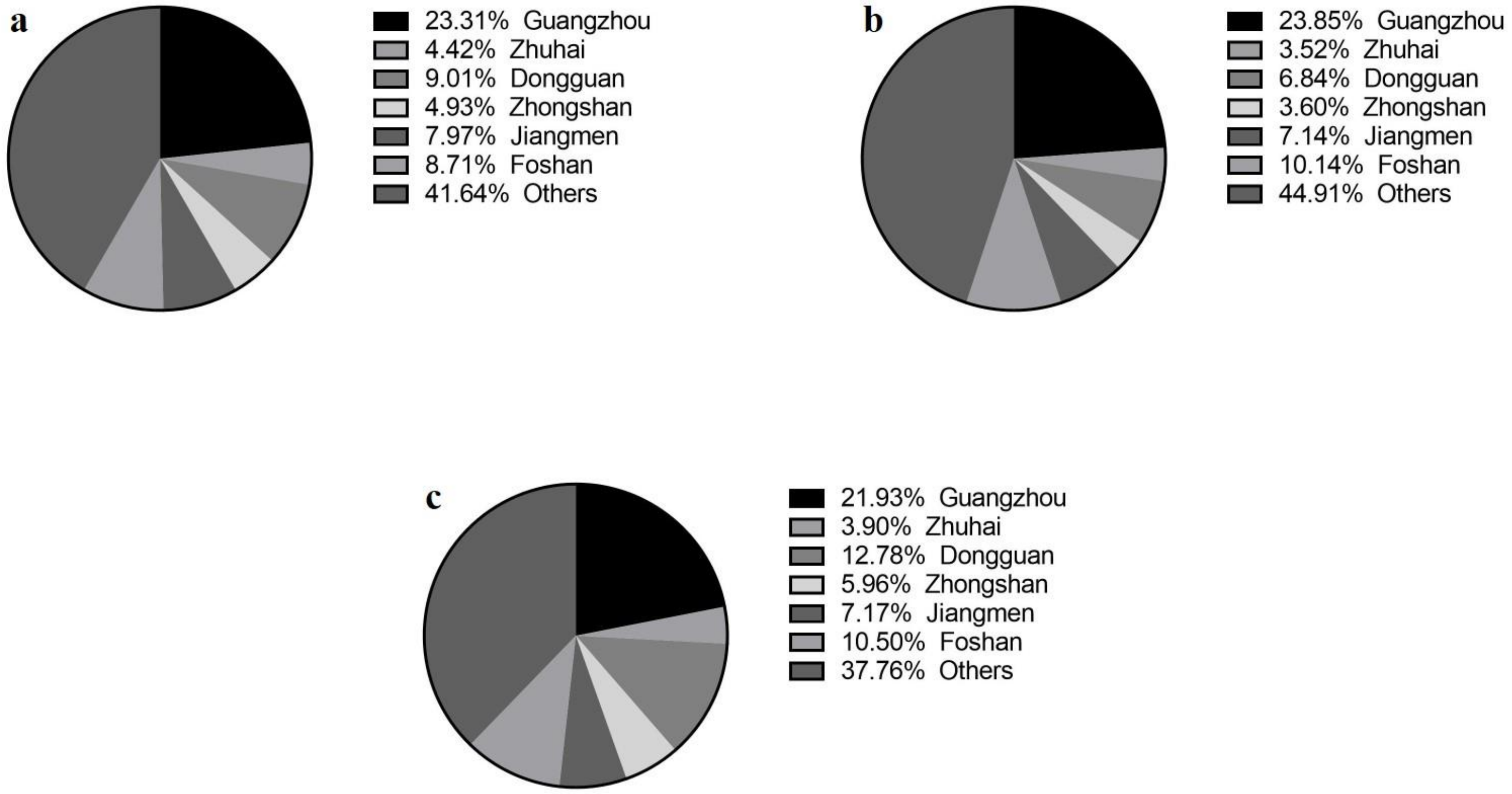

Figure S1. Proportions of light industry (a), heavy industry (b), and plastic and rubber industry (c) among six cities.

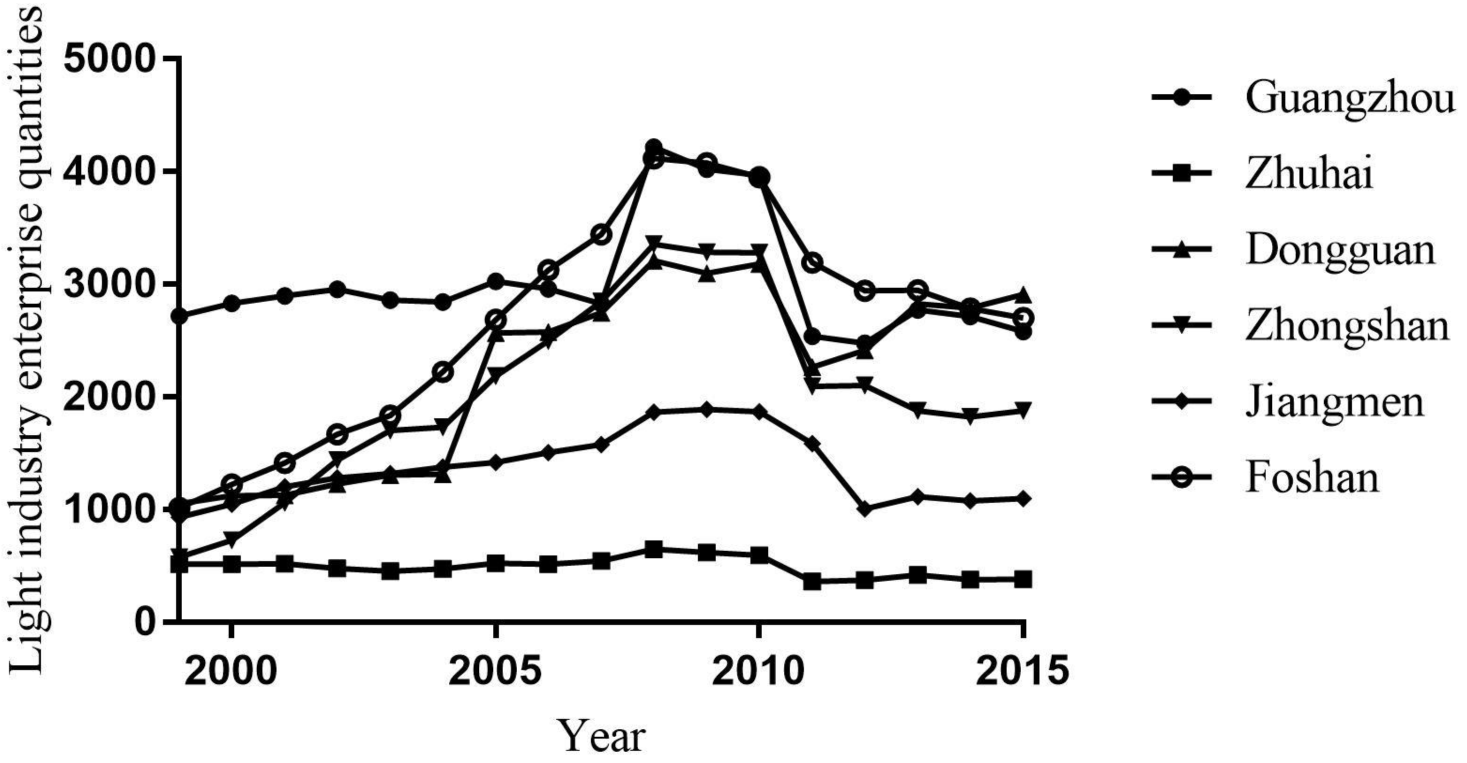

Figure S2. Trends of light industry enterprise quantity among six cities.

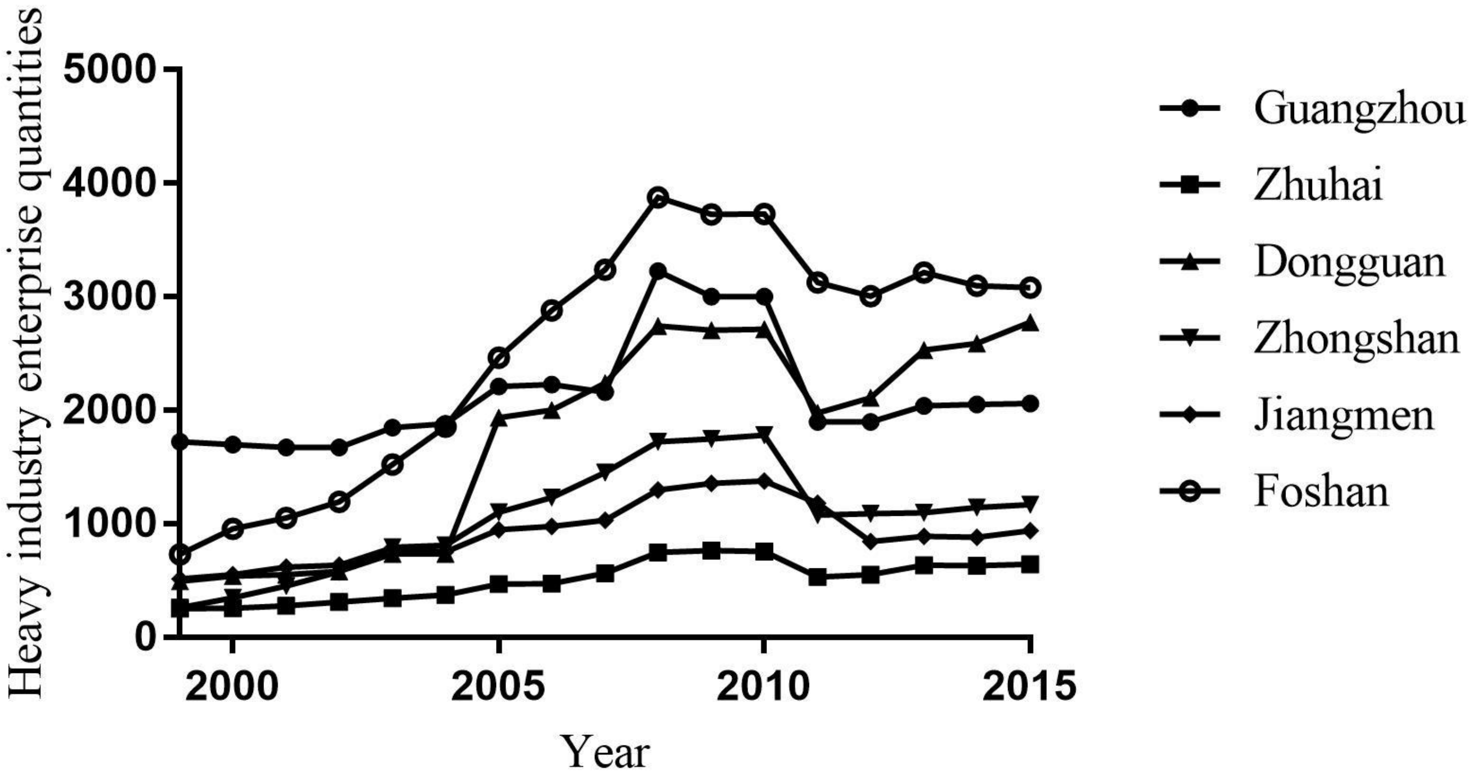

Figure S3. Trends of heavy industry enterprise quantity among six cities.

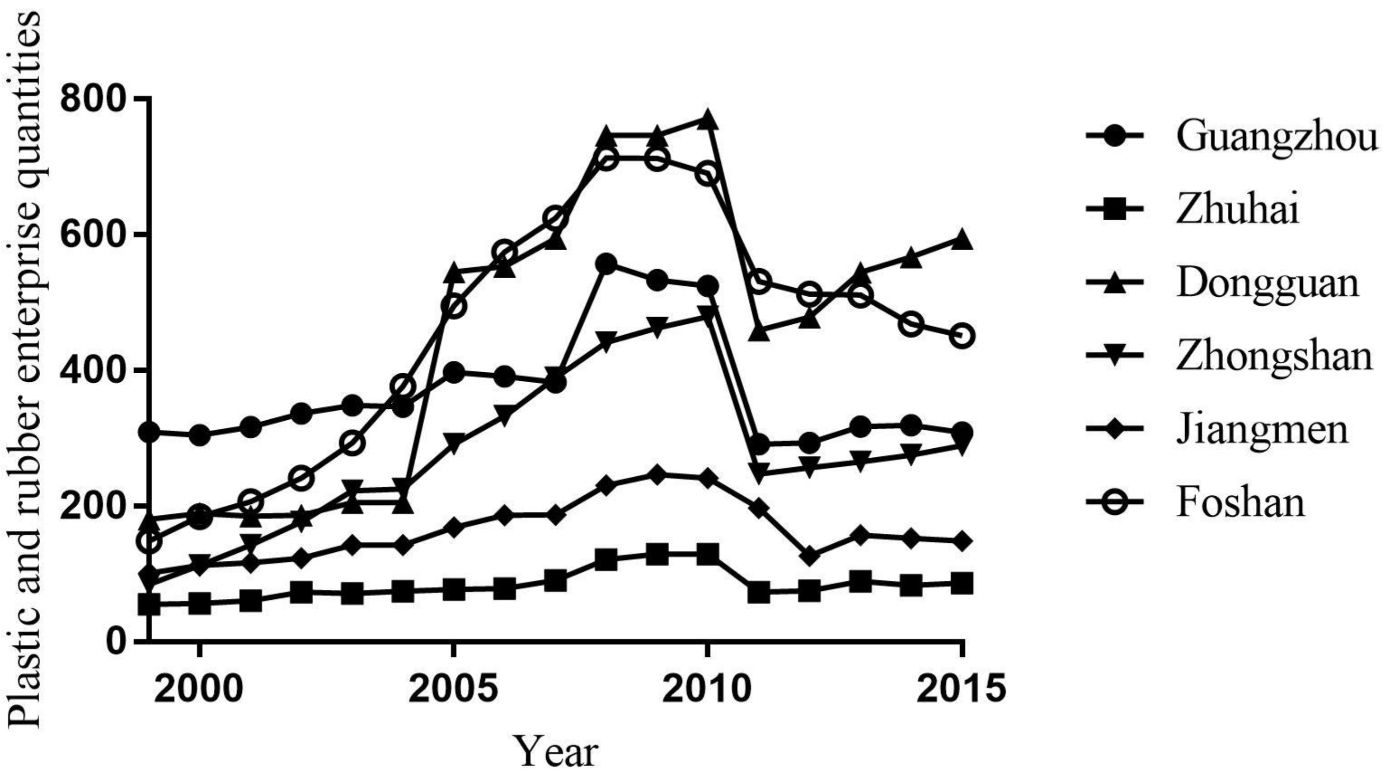

Figure S4. Trends of plastic and rubber industry enterprise quantity among six cities

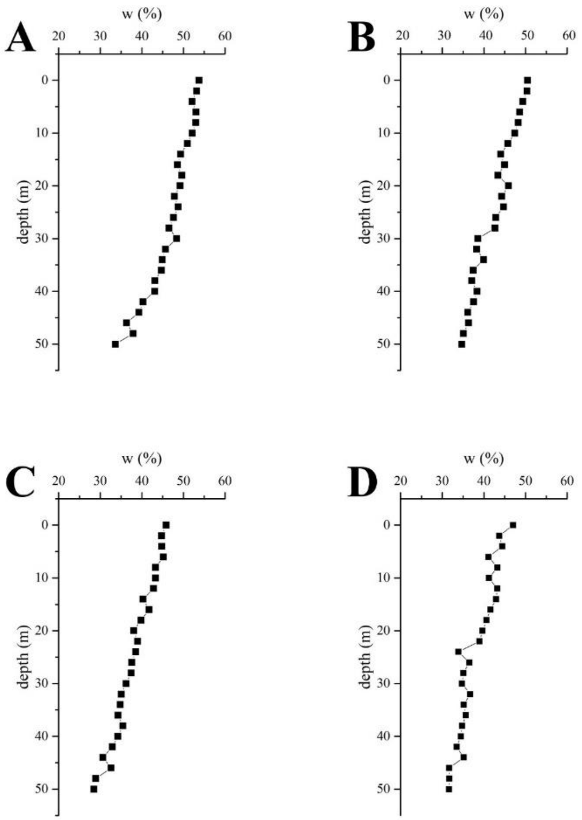

Figure S5. Profiles of water content in the sediment.
